# Defective FANCI Binding by a Fanconi Anemia-Related FANCD2 Mutant

**DOI:** 10.1371/journal.pone.0114752

**Published:** 2014-12-09

**Authors:** Koichi Sato, Masamichi Ishiai, Minoru Takata, Hitoshi Kurumizaka

**Affiliations:** 1 Laboratory of Structural Biology, Graduate School of Advanced Science & Engineering, Waseda University, Tokyo, Japan; 2 Laboratory of DNA Damage Signaling, Department of Late Effects Studies, Radiation Biology Center, Kyoto University, Kyoto, Japan; The University of Hong Kong, Hong Kong

## Abstract

FANCD2 is a product of one of the genes associated with Fanconi anemia (FA), a rare recessive disease characterized by bone marrow failure, skeletal malformations, developmental defects, and cancer predisposition. FANCD2 forms a complex with FANCI (ID complex) and is monoubiquitinated, which facilitates the downstream interstrand crosslink (ICL) repair steps, such as ICL unhooking and nucleolytic end resection. In the present study, we focused on the chicken FANCD2 (cFANCD2) mutant harboring the Leu234 to Arg (L234R) substitution. cFANCD2 L234R corresponds to the human FANCD2 L231R mutation identified in an FA patient. We found that cFANCD2 L234R did not complement the defective ICL repair in FANCD2^−/−^ DT40 cells. Purified cFANCD2 L234R did not bind to chicken FANCI, and its monoubiquitination was significantly deficient, probably due to the abnormal ID complex formation. In addition, the histone chaperone activity of cFANCD2 L234R was also defective. These findings may explain some aspects of Fanconi anemia pathogenesis by a FANCD2 missense mutation.

## Introduction

Fanconi anemia (FA) is a rare recessive disease, with symptoms including bone marrow failure, skeletal malformations, developmental defects, and cancer predisposition [1]. Cells from FA patients are hypersensitive to interstrand DNA crosslinking (ICL) reagents, which prevent the progression of replication forks by covalent-crosslinking between complementary strands [2]–[4]. Therefore, the FA causative genes are considered to constitute the ICL repair pathway, which is called the FA pathway [5]–[9].

Sixteen genes have been identified as FA causative genes, and all are conserved in higher eukaryotes except for FANCM and FANCP, which are found even in yeast species [3]. Among these sixteen genes, two FA proteins, FANCI and FANCD2, form a stoichiometric complex called the ID complex [10]–[14]. Eight FA proteins, FANCA, -B, -C, -E, -F, -G, -L, and –M, form another complex (FA core complex) together with five FA-associated proteins (FAAP20, FAAP24, FAAP100, MHF1, and MHF2) [15]–[29]. During the ICL repair processes, the ID and FA core complexes promptly accumulate at the ICL sites in chromosomes [10], [11], [30], [31], and promote ICL repair with the other FA-related proteins [32]–[39].

The FA core complex, which contains a ubiquitin E3 ligase subunit (FANCL), then monoubiquitinates both the FANCI and FANCD2 subunits of the ID complex [10], [11], [40]–[43]. In particular, monoubiquitinated FANCD2 plays an essential role in the recruitment of downstream nucleases, which remove the bases with ICL [7], [34]–[37], [44]–[47]. The ID complex preferentially binds to branched DNA *in vitro*, and the monoubiquitination of FANCD2 in the ID complex is robustly enhanced in the presence of DNA [12], [48]–[50]. FANCD2 is site-specifically monoubiquitinated by FANCL [30], [40], [41], [48]–[50], and cells with a mutation of the targeted FANCD2 Lys residue are remarkably defective in the ICL repair [30], [51]. This fact indicates that the FANCD2 monoubiquitination is essential for the ICL repair by the FA pathway.

In addition, FANCD2 possesses histone chaperone activity, which modifies the chromatin structure by promoting histone deposition/eviction around the ICL sites [14]. The chicken FANCD2 R305W mutant (cFANCD2 R305W), in which Arg305 is replaced by Trp, is specifically defective in the histone chaperone activity *in vitro* and *in vivo*, and complements the ICL repair-defective phenotype of the FANCD2^−/−^ DT40 cells with a significantly reduced rate [14]. These results revealed that the histone chaperone activity of FANCD2 may play an important role during ICL repair, probably by modifying the chromatin structure to allow access for the proteins required for the downstream steps of the FA pathway [5], [6], [14]. Importantly, the human FANCD2 R302W mutation, which corresponds to the chicken FANCD2 R305W mutation, has been identified as an FA causative mutation in a patient [52].

A number of FANCD2 mutations, which are generally considered to reduce protein stability, have been identified in FA patients [52]–[54]. However, the means by which the residual FANCD2 protein functions participate in the ICL repair remain poorly understood. In the present study, we focused on the chicken FANCD2 L234R (cFANCD2 L234R) mutation, which corresponds to the human FANCD2 missense mutation at the Leu231 residue, found in an FA patient [53].

## Materials and Methods

### Generation of DT40 cell lines expressing chicken FANCD2 mutants

For the construction of the cFANCD2 fusions, the cDNA encoding FANCD2 was inserted into the GFP or the histone H2B-GFP expression vector, using the Gateway system (Invitrogen). To establish stable cell lines expressing the cFANCD2 fusions, the plasmids were transfected into FANCD2^−/−^ DT40 cells [55]. The DT40 clones expressing the cFANCD2-mutant fusions were selected by the previously described method [56], [57].

### Cisplatin sensitivity assay

The cisplatin sensitivity of the DT40 cell lines was assessed by colony formation, in medium containing the indicated amount of cisplatin (Nihon-Kayaku) and 1.4% methylcellulose, as previously described [56], [57].

### Detection of FANCD2 and FANCI proteins in cell extracts

The chicken DT40 cells were treated with MMC (500 ng/ml for 6 h) or without MMC, and the chromatin fraction was obtained by the method described previously [51], [58]. The GFP-cFANCD2 proteins produced in the DT40 cells were fractionated by 6% SDS–PAGE. The ubiquitinated or non-ubiquitinated cFANCD2 and cFANCI proteins were detected by western blotting with the anti-chicken FANCD2 and FANCI antibodies, respectively.

### Immunoprecipitation

The chicken DT40 cells producing the GFP-cFANCD2 proteins were lysed in lysis buffer, containing 20 mM Tris-HCl (pH 7.5), 150 mM NaCl, 0.5% NP-40, 1 mM phenylmethylsulfonyl fluoride, 5 mM NaF, and protease inhibitor cocktail (Roche). Afterward, the lysates were treated with 50 unit/mL of Benzonase, and were mixed with anti-GFP beads (MBL). After three washes with lysis buffer, the immunoprecipitates were analyzed by western blotting using anti-chicken FANCD2 and cFANCI antibodies.

### Purification of chicken FANCI, FANCD2, FANCL and human UBE2T

Wild type cFANCD2 and chicken FANCI (cFANCI) were expressed as His_6_-tag fused proteins in the *Escherichia coli* BL21(DE3) codon(+)RIL strain (Stratagene), and were purified as previously described [14], [48]. The Leu234 to Arg mutation in the cFANCD2 cDNA was generated by site directed mutagenesis, using the following primers: 3′-gtagc tgctt actga aacag ggcag acggc-5′ and 3′-gctct ctggc aacct cattt tgctg gg-5′. The purification of the cFANCD2 L234R mutant and other cFANCD2 mutants was performed by the same method as for wild type cFANCD2.

The purification of chicken FANCL (cFANCL) and human UBE2T (hUBE2T) was performed by the previously described methods [14], [48]. Each protein was expressed as a GST-tagged protein in the *Escherichia coli* BL21(DE3) codon(+)RIL strain. The GST-tag was removed from hUBE2T by PreScission protease digestion during the purification steps. The concentrations of the purified proteins were assayed by the Bradford method [59], using bovine serum albumin as the standard protein.

### Purification of recombinant core histones

Human core histones were purified by the same method described previously [60], [61]. Human histones H2A, H2B, and H3 were each overexpressed in *Escherichia coli* BL21(DE3) cells, and human histone H4 was overexpressed in *Escherichia coli* JM109(DE3) cells. All core histones were expressed as His_6_-tagged proteins. The histone H3/H4 and H2A/H2B complexes were each formed by the salt dialysis method, and were purified as described previously [62], [63]. The His_6_-tag was removed during the purification, using thrombin protease.

### Gel-filtration assay

The purified cFANCD2, cFANCI, and equimolar mixture of cFANCD2 and cFANCI were subjected to Superdex 200 10/300 GL gel filtration column chromatography (GE Healthcare), in buffer containing 20 mM Tris-HCl (pH 8.0), 10% glycerol, 200 mM NaCl, and 5 mM 2-mercaptoethanol. The elution profiles of the purified proteins were monitored by UV absorption at 280 nm. The fractions at 7.5–14.5 mL of elution volume were separated by 7% SDS-PAGE, and the proteins were detected by silver staining.

### Monoubiquitination assay

The monoubiquitination assay was performed as previously described [48]. Purified cFANCD2 (1 µM) and cFANCI (1 µM) were mixed with HA-tagged ubiquitin (10 µM) (Boston Biochem), recombinant human E1 (75 nM) (Boston Biochem), hUBE2T (8 µM), GST-cFANCL (2 µM), and 49 bp dsDNA (100 µM) in 10 µL of reaction buffer, containing 50 mM Tris-HCl (pH 7.5), 4% glycerol, 64 mM NaCl, 2 mM ATP, 2 mM MgCl_2_, and 0.5 mM dithiothreitol. The reaction mixtures were incubated for 90 min at 30°C. The reactions were then stopped by the addition of 10 µL of 2-fold SDS buffer, containing 125 mM Tris-HCl (pH 6.8), 4% SDS, 20% glycerol, 0.01% bromophenol blue, and 0.2 M dithiothreitol. The samples were separated by 7% SDS-PAGE, and the ubiquitinated products were visualized by western blotting using an anti-HA antibody (F-7; Santa Cruz Biotechnology, Inc.) and by Coomassie Brilliant Blue staining. The amounts of monoubiquitinated FANCD2 were quantitated by LAS4000 using Multi Gauge software (Fujifilm).

### Supercoiling assay for nucleosome formation

The supercoiling assay for nucleosome assembly was performed as previously described [14]. Before the nucleosome assembly reaction, the H2A/H2B complex (85 ng) and the H3/H4 complex (85 ng) were preincubated with cFANCD2 in the presence or absence of cFANCI at 37°C for 15 min, in 9 µL of reaction buffer, containing 18 mM Tris-HCl (pH 8.0), 67 mM NaCl, 2.2 mM MgCl_2_, 2.8 mM dithiothreitol, and 3.3% glycerol. To start the nucleosome assembly reaction, 100 ng of relaxed circular φX174 dsDNA, which was preincubated with 2 units of wheat germ topoisomerase I (Promega) at 37°C for 150 min, in buffer containing 18 mM Tris-HCl (pH 8.0), 8% glycerol, 84 mM NaCl, 2 mM MgCl_2_, 0.2 mM EDTA, and 2.7 mM dithiothreitol, was added to the reaction. The reaction was continued at 37°C for 60 min. Afterwards, the reaction mixture was deproteinized with 60 µL of a proteinase K solution, containing 20 mM Tris-HCl (pH 8.0), 20 mM EDTA, 0.5% SDS, and 0.5 mg/mL proteinase K. The DNA was then extracted with phenol-chloroform, and subsequently precipitated with ethanol. Topoisomers were separated by 1% agarose electrophoresis in TAE buffer (40 mM Tris-acetate and 1 mM EDTA), and were visualized by SYBR Gold (Invitrogen) staining.

## Results

### The cFANCD2 L234R mutant is defective in ICL repair in DT40 cells

**Figure 1:**
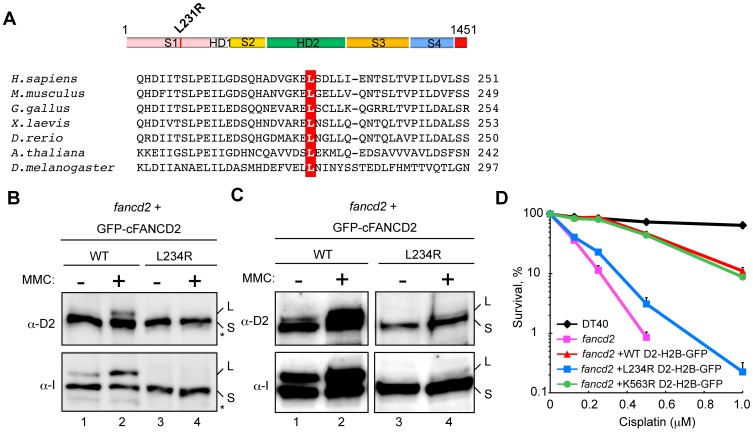
FANCD2^−/−^ DT40 cells expressing the chicken FANCD2 L234R mutant are defective in ICL repair. (A) Schematic representation of human FANCD2. S1, HD1, S2, HD2, S3, and S4 denoted on the bar indicate the FANCD2 subdomains, solenoid 1, helical domain 1, solenoid 2, helical domain 2, solenoid 3, and solenoid 4, respectively. The FANCD2 C-terminal acidic region is colored red. The primary structure of the N-terminal regions of *Homo sapiens*, *Mus musculus*, *Gallus gallus*, *Xenopus laevis*, *Danio rerio*, *Arabidopsis thaliana*, and *Drosophila melanogaster* FANCD2 are aligned. The residues corresponding to human FANCD2 L231 are colored red. (B) ID complex monoubiquitination in cFANCD2^−/−^ DT40 cells expressing the GFP-wild-type (WT) cFANCD2 or cFANCD2 L234R. Cells were treated with or without mitomycin C (MMC), and whole-cell extracts were analyzed by western blotting using anti-cFANCD2 (α-D2) and anti-cFANCI (α-I) antibodies. S-forms and L-forms indicate non-ubiquitinated and monoubiquitinated forms of cFANCD2 and cFANCI, respectively. Non-specific bands are marked by asterisks. (C) ID complex monoubiquitination in the chromatin fractions from cFANCD2^−/−^ DT40 cells expressing GFP-WT cFANCD2 or cFANCD2 L234R. Cells were treated with or without MMC, and chromatin fractions were analyzed as in panel (B). (D) Cisplatin sensitivity assay of the cFANCD2^−/−^ DT40 cells expressing H2B-GFP fusions with the WT cFANCD2, cFANCD2 K563R, or cFANCD2 L234R. The mean values are shown with s.d. from triplicate measurements.

The human FANCD2 Leu231 residue, which is mutated to Arg in an FA patient [53], is evolutionarily conserved, and corresponds to the chicken FANCD2 (cFANCD2) Leu234 residue (Fig. 1A). To study the effect of the Leu to Arg substitution on the ICL repair, we performed the complementation assay with the FANCD2^−/−^ chicken DT40 cells producing the cFANCD2 L234R mutant, in which the Leu234 residue was replaced by Arg. We found that the cFANCD2 L234R mutant was not monoubiquitinated in the whole cell extracts, unlike wild-type cFANCD2 (Fig. 1B and S1A Figure). A trace amount of the cFANCD2 L234R monoubiquitination was detected in the chromatin fraction, but it was quite low, as compared to wild type cFANCD2 (Fig. 1C and S1C Figure). The cFANCI monoubiquitination, which strictly requires the FANCD2 monoubiquitination [58], was also clearly defective in the cFANCD2^−/−^ DT40 cells producing cFANCD2 L234R (Fig. 1B, C, S1B and S1D Figure).

Previous studies established that monoubiquitination is a prerequisite for the chromatin localization of FANCD2, and hence the DNA repair activities. Exogenously produced cFANCD2, when expressed as a fusion protein with histone H2B in DT40 cells, is targeted to chromatin, and the cFANCD2-H2B fusion protein complements the ICL-deficient phenotype of FANCD2^−/−^ DT40 cells [51]. To clarify the function of the FANCD2 L234R mutant further, we expressed the cFANCD2 proteins as fusions with histone H2B in the FANCD2^−/−^ DT40 cells. Consistent with the previous report [51], cFANCD2-H2B and cFANCD2 K563R-H2B rescued the cisplatin-sensitive phenotype of the FANCD2^−/−^ DT40 cells (Fig. 1D). The cFANCD2 K563R mutant, in which the Lys563 residue that is monoubiquitinated in cFANCD2 is replaced by Arg, is reportedly defective in the monoubiquitination, but is targeted to chromatin by an H2B fusion [51]. In contrast, we found that cFANCD2 L234R-H2B did not complement the cisplatin-sensitive phenotype of the cFANCD2^−/−^ DT40 cells (Fig. 1D). These results suggested that the cFANCD2 L234R mutant is defective in the ICL repair even if targeted to chromatin.

### Defective monoubiquitination of cFANCD2 L234R *in vitro*

**Figure 2:**
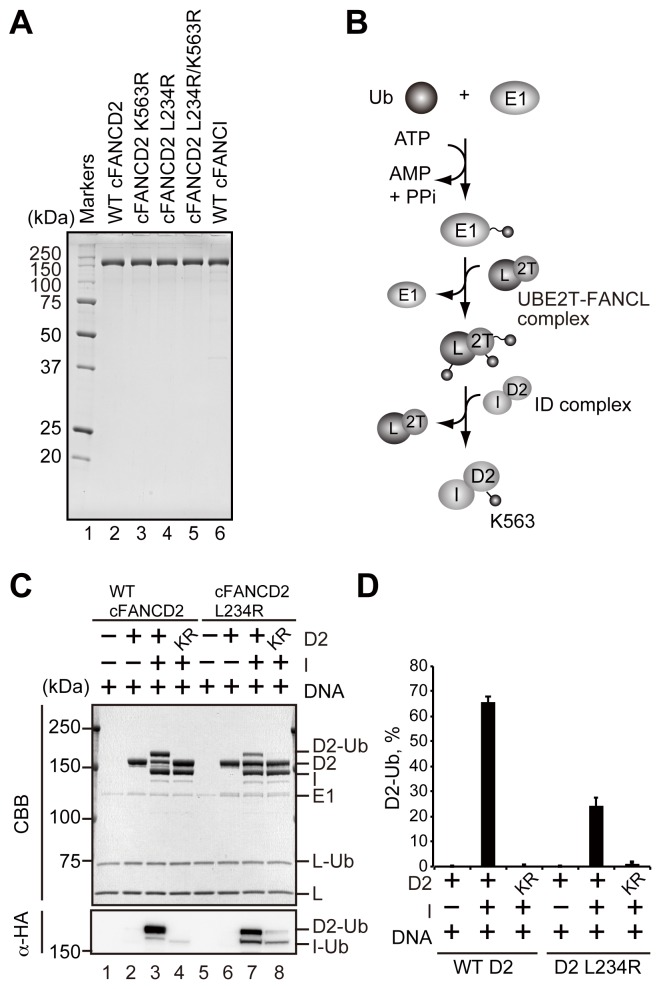
Chicken FANCD2 L234R is defective in monoubiquitination *in vitro*. (A) Purified cFANCD2 and cFANCI proteins used for *in vitro* assays were analyzed by 15% SDS-PAGE with Coomassie Brilliant Blue staining. Lane 1 indicates the molecular mass markers. Lanes 2–5 indicate purified wild type (WT) cFANCD2, cFANCD2 K563R, cFANCD2 L234R, and cFANCD2 L234R/K563R, respectively. Lane 6 indicates purified WT cFANCI. (B) Schematic diagram of the monoubiquitination assay. The monoubiquitination assay was performed by incubating cFANCD2 (D2) or cFANCI (I)-cFANCD2 with HA-tagged ubiquitin (Ub), human E1, human UBE2T (2T) and GST-cFANCL (L) in the presence of ATP. The high-energy thioester bond between ubiquitin and the cysteine residue of the substrate is indicated as a wavy line. The stable covalent bond between ubiquitin and the lysine residue of the substrate is indicated as a linear line. (C) The monoubiquitination assay was performed in the presence of 100 µM dsDNA. Ubiquitinated proteins were analyzed by 7% SDS-PAGE, and were detected by anti-HA antibody (α-HA) and Coomassie Brilliant Blue staining. Lane 1 indicates a control experiment without WT cFANCD2 and cFANCI. Lane 2 indicates an experiment with WT cFANCD2 in the absence of cFANCI. Lanes 3 and 4 indicate experiments with WT cFANCD2 and cFANCD2 K563R in the presence of WT cFANCI, respectively. Lanes 5–8 indicate experiments with cFANCD2 L234R and cFANCD2 L234R/K563R, instead of WT cFANCD2 and cFANCD2 K563R. (D) Graphical representation of the experiments shown in C, lanes 2–4 and lanes 6–8. The mount of monoubiuqitinated FANCD2 was quantitated. Means of three independent experiments are shown with s.d.’s.

We then purified the cFANCD2, cFANCD2 K563R, cFANCD2 L234R, cFANCD2 L234R/K563R, and cFANCI proteins, as bacterially produced recombinant proteins, and performed the *in vitro* monoubiquitination assay (Fig. 2A). In this assay, purified cFANCD2 and cFANCI were incubated with HA-tagged ubiquitin, E1, UBE2T (E2), and FANCL (E3 ligase) in the presence of ATP and Mg^2+^ (Fig. 2B). The FANCD2 monoubiquitination is reportedly stimulated in the presence of DNA [47]–[49], and thus the monoubiquitination reaction included DNA. The ubiquitinated proteins were then fractionated on SDS-polyacrylamide gels, and detected by staining with Coomassie Brilliant Blue and western blotting with an anti-HA antibody detecting HA-tagged ubiquitin.

Consistent with previous studies [48]–[50], the monoubiquitination of wild type cFANCD2 was robustly stimulated in the presence of cFANCI (Fig. 2C, lanes 2 and 3, and D), indicating that the ID complex formation is required for the efficient monoubiquitination of cFANCD2. The monoubiquitination of the cFANCD2 K563R mutant was not detected in the presence of cFANCI (Fig. 2C, lane 4, and D). This confirmed that the cFANCD2 monoubiquitination specifically occurs on the Lys563 residue. On the other hand, in the cFANCD2 L234R mutant, the Lys563-specific monoubiquitination was also detected, but the level was quite low, even in the presence of cFANCI (Fig. 2C, lanes 5–8, and D). Therefore, consistent with the *in vivo* results (Fig. 1B), cFANCD2 L234R may be defective in site-specific monoubiquitination.

### cFANCD2 L234R is defective in the complex formation with FANCI

**Figure 3:**
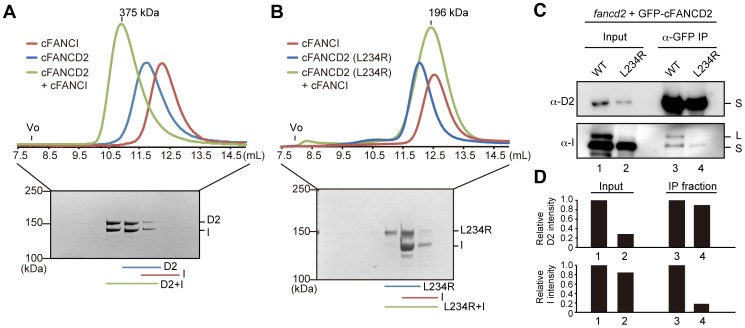
Chicken FANCD2 L234R is defective in FANCI binding. (A, B) Gel filtration analysis of the ID complex formation. cFANCD2, cFANCI, and the mixture of cFANCD2 and cFANCI were fractionated on the Superdex 200 gel filtration column. The SDS-PAGE analysis of the mixture of the cFANCD2 and FANCI fractions is shown below the gel filtration profiles. The void volume of the Superdex200 column is indicated as ‘Vo’ on the gel filtration profiles. Experiments with wild type (WT) cFANCD2 and WT cFANCI (A), cFANCD2 L234R and WT cFANCI (B). (C) Immunoprecipitation analysis of the cFANCD2 and cFANCI interaction. The cell extracts from chicken DT40 cells expressing GFP-WT cFANCD2 or cFANCD2 L234R were immunoprecipitated with an anti-GFP antibody. The immunoprecipitates were separated by 6% SDS-PAGE, and cFANCD2 and cFANCI were detected by western blotting using anti-chicken FANCD2 and FANCI antibodies, respectively. (D) Graphic representation of the relative intensity of the bands corresponding to GFP-cFANCD2 (upper) and endogenous cFANCI (lower) shown in panel (C). The band intensity was normalized relative to that of cFANCD2 or cFANCI.

Previous biochemical studies revealed that the ID complex formation is essential for the site-specific monoubiquitination of FANCD2 [41], [48]–[50]. We then tested whether the cFANCD2 L234R mutation affected the ID complex formation. During ICL repair, FANCD2 directly binds to FANCI, and forms a stoichiometric complex, called the ID complex [10]–[14]. A gel filtration analysis revealed that the purified cFANCD2 and cFANCI proteins formed the ID complex, which eluted as a single peak corresponding to an apparent molecular weight of 375 kDa (Fig. 3A and S2A Figure). This elution profile of the ID complex was different from those of the cFANCI and cFANCD2 proteins alone (Fig. 3A and S2A Figure). Interestingly, we found that an equimolar mixture of cFANCD2 L234R and cFANCI resulted a peak corresponding to 196 kDa, which covered the regions of the two peaks of cFANCD2 L234R and cFANCI alone (Fig. 3B and S2B). In fact, cFANCD2 L234R and cFANCI separately eluted from the gel filtration column, and only a few fractions contained both proteins (Fig. 3B, bottom panel, and S2B Figure). Interestingly, a cell-based pull-down assay revealed that cFANCD2 L234R bound to cFANCI with substantially reduced efficiency, as compared to wild type cFANCD2 (Fig. 3C, S2C and S2DFigure), suggesting its defective ID complex formation *in vivo*. In this assay, the amount of the input GFP-cFANCD2 L234R was lower than that of wild type GFP-cFANCD2, although the amount of cFANCI was not significantly affected (Fig. 3C and D). This may be due to the fact that the defective ID complex formation destabilized cFANCD2 L234R in cells. However, similar amounts of GFP-cFANCD2 L234R and GFP-cFANCD2 were obtained in the immunoprecipitated (IP) fractions under our experimental conditions (Fig. 3C and D). We therefore compared the cFANCI binding by pull-down assays with similar amounts of GFP-cFANCD2 L234R and wild type GFP-cFANCD2 in the IP fraction. In addition, cFANCD2 L234R did not support the cFANCI monoubiquitination, which occurs in the ID complex (Fig. 1C, 3C and S2D Figure). Consistently, the cFANCD2 monoubiquitination, which also occurs in the ID complex, was markedly defective *in vivo* (Fig. 1B and C) and *in vitro* (Fig. 2C and D). These data strongly suggested that, unlike wild type cFANCD2, cFANCD2 L234R does not properly form the ID complex with cFANCI.

### Reduced histone chaperone activity of cFANCD2 L234R

**Figure 4:**
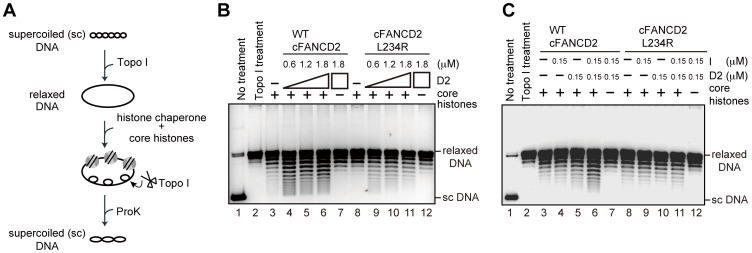
Chicken FANCD2 L234R exhibits impaired histone chaperone activity. (A) Schematic diagram of the supercoiling assay for nucleosome formation. Core histones were assembled on the relaxed plasmid DNA by a histone chaperone in the presence of wheat germ topoisomerase I. After deproteinization, the topoisomers were separated by agarose gel electrophoresis. (B) Supercoiling assays for nucleosome formation were performed with wild type (WT) cFANCD2 (lanes 4–7) and cFANCD2 L234R (lanes 9–12). Protein concentrations were 0 (lanes 3 and 8), 0.6 (lanes 4 and 9), 1.2 (lanes 5 and 10), and 1.8 µM (lanes 6, 7, 11, and 12). Highly supercoiled DNA is denoted as ‘sc DNA’. (C) Supercoiling assays for nucleosome formation were performed with WT cFANCD2 or cFANCD2 L234R, in the presence of WT cFANCI. The protein concentrations used in the assay are indicated at the top of the panel.

We previously reported that FANCD2 possesses histone chaperone activity, which mediates the incorporation/eviction of histones in chromatin, and this activity is actually important for ICL repair [14]. To evaluate the histone chaperone activity of cFANCD2 L234R, we employed the supercoiling assay. In this assay, nucleosome assembly with histones H2A, H2B, H3, and H4 was mediated by cFANCD2 on relaxed plasmid DNA. Nucleosome formation can be detected as topological changes of the plasmid DNA in the presence of topoisomerase I (Fig. 4A). Consistent with our previous results [14], wild type cFANCD2 exhibited the histone chaperone activity (Fig. 4B, lanes 4–7). In contrast, the histone chaperone activity of cFANCD2 L234R was clearly reduced, as compared with that of wild type cFANCD2 (Fig. 4B, lanes 9–12).

Since the nucleosome assembly activity of FANCD2 is reportedly stimulated by FANCI [14], we next performed the supercoiling assay in the presence of FANCI. As shown in Fig. 4C (lane 5), the histone chaperone activity of cFANCD2 was not detected under the low cFANCD2 conditions (0.15 µM). Consistent with the previous results, the histone chaperone activity of cFANCD2 was significantly stimulated in the presence of a stoichiometric amount of cFANCI (0.15 µM) (Fig. 4C, lane 6). However, we found that the nucleosome assembly activity of cFANCD2 L234R was not stimulated by cFANCI (Fig. 4C, lane 11). This may be due to the defective formation of the ID complex between cFANCD2 L234R and cFANCI (Fig. 3).

## Discussion

**Figure 5:**
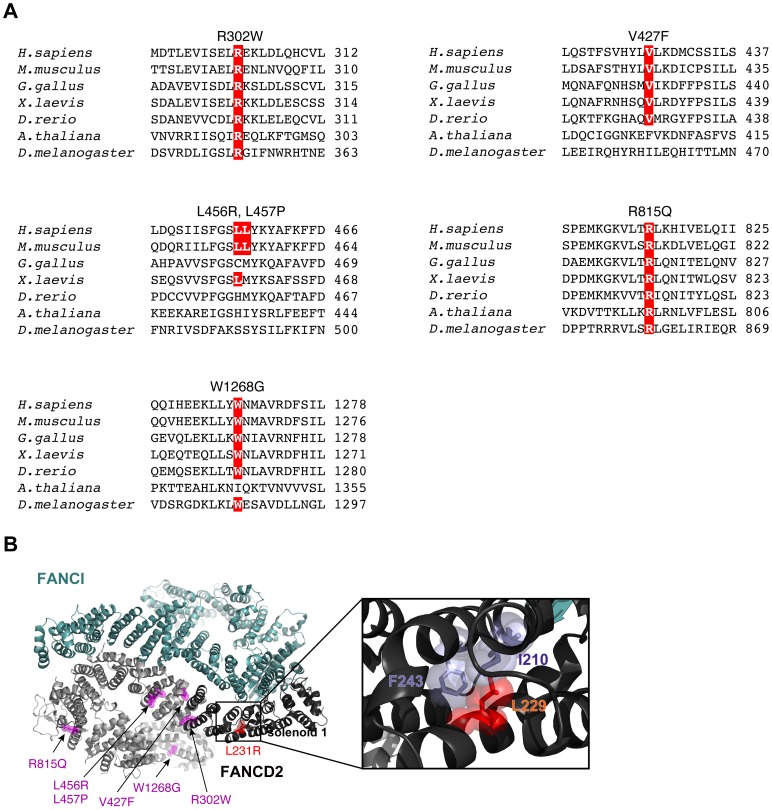
The location of the FANCD2 Leu229 residue in the mouse ID complex structure (3S4W). (A) Amino acid sequences of *Homo sapiens* FANCD2 surrounding the FA-related missense mutations. Alignment of the FANCD2 sequences of *Homo sapiens, Mus musculus*, *Gallus gallus*, *Xenopus laevis*, *Danio rerio*, *Arabidopsis thaliana*, and *Drosophila melanogaster*. The residues corresponding to human FANCD2 R302, V427, L456, L457, Q815, and W1268, which are mutated in the FA patients, are colored red. (B) The FANCD2 and FANCI proteins are colored grey and green, respectively. The mouse FANCD2 solenoid 1 and the FANCD2 Leu229 residue, corresponding to the cFANCD2 L234 and human FANCD2 L231 residues, are colored black and red, respectively. The FA-associated residues are colored magenta. Ile210 and Phe243 of mouse FANCD2, which are located adjacent to mouse FANCD2 Leu229, are also colored light blue. The van der Waals surfaces of the FANCD2 Ile210, Leu229 and Phe243 side chain atoms are represented.

FANCD2 is considered to be a key player in ICL repair by the FA pathway [2]–[9]. Among FA patients, seven missense mutations have been found in the FANCD2 gene [52]–[54]. Each of these human FANCD2 mutants contains a single amino acid substitution, at Leu231 to Arg (L231R), Arg302 to Trp (R302W), Val427 to Phe (V427F), Leu456 to Arg (L456R), Leu457 to Pro (L457P), Arg815 to Gln (R815Q), or Trp1268 to Gly (W1268G). Among them, L231, R302, and R815 are highly conserved among the mammalian, chicken, frog, fish, and plant proteins (Figs. 1A and 5A). V427 and W1268 are not conserved in the plant protein (Fig. 5A). On the other hand, L456 and L457 are not evolutionally conserved (Fig. 5A). These residues were mapped on the crystal structure of the ID complex (Fig. 5B) [13].

It remains largely unknown whether these mutations affect the FANCD2 function *per se*. In the present study, we focused on the Leu231 to Arg mutation. The Leu231 residue in human FANCD2 is conserved as the Leu234 residue in the chicken FANCD2 protein (Fig. 1A). We found that cFANCD2 L234R did not complement the ICL repair-deficient phenotype of the FANCD2^−/−^ DT40 cells (Fig. 1D), and was defective in the monoubiquitination of cFANCD2 and cFANCI (Fig. 1B and C). These *in vivo* results suggest that the FANCD2^−/−^ DT40 cells with the cFANCD2 L234R mutant reflect the characteristics of the FA patient cells. We then purified the cFANCD2 L234R protein, and performed biochemical analyses. Interestingly, we found that cFANCD2 L234R is clearly defective in the ID complex formation with cFANCI (Fig. 3A and B). Intriguingly, a cell-based pull-down assay also revealed that cFANCD2 is defective in the cFANCI binding *in vivo* (Fig. 3C). Although the original report did not address the stability of the FANCD2 protein bearing this mutation, the level of the protein might be reduced (as found with the other FANCD2 missense mutations) because of the lack of the FANCI interaction [11], [53].

In the ID complex, both FANCD2 and FANCI are site-specifically monoubiquitinated by the FA core complex containing other essential FA proteins [10], [11], [30]. The FANCD2 monoubiquitination is especially important, because a FANCD2 mutant, in which its target Lys residue is mutated, is defective in chromatin localization, and hence also in ICL repair [30], [51]. On the other hand, the mutation of FANCI on its target Lys residue may not substantially affect the FA repair pathway [58]. However, proper FANCD2 monoubiquitination strictly requires FANCI [10], [11], [41], [48]–[50], [58]. These facts suggest that the correct FANCD2 monoubiquitination that occurs in the ID complex plays an essential role in ICL repair by the FA pathway. Consistently, we found that cFANCD2 L234R, which exhibits impaired cFANCI binding, is also markedly defective in its monoubiquitination both *in vivo* and *in vitro* (Figs. 1B and 2). In addition, the FANCD2 monoubiquitination may function to recruit the other FA proteins, such as the SLX4-XPF (FANCP-FANCQ) nuclease complex, to the damaged chromatin site [44]–[47]. The reduced FANCD2 monoubiquitination due to the defective ID complex formation may be responsible for producing a major pathogenic effect in FA patients with the FANCD2 Leu231 mutation.

Our gel filtration and pull-down analyses revealed that cFANCD2 L234R was defective in the ID complex formation (Fig. 3). These results suggest that the Leu234 residue of cFANCD2 may be directly involved in binding to cFANCI. However, in the crystal structure of the ID complex containing mouse FANCD2 and FANCI, the mouse FANCD2 Leu229 residue, corresponding to cFANCD2 Leu234, is not located on the binding surface with FANCI (Fig. 5B) [13]. The Leu229 residue is buried within the solenoid 1 domain of FANCD2, and the Leu (hydrophobic) to Arg (hydrophilic) replacement may drastically change the hydrophobic/hydrophilic environment around the residue within this domain (Fig. 5B, right panel). Therefore, the cFANCD2 L234R mutation may indirectly cause the defective formation of the ID complex, probably through the structural change of solenoid 1.

We previously reported that the human and chicken FANCD2 proteins possess histone chaperone activity, and the chicken FANCD2 R305W mutant (cFANCD2 R305W), which corresponds to the R302W mutant of human FANCD2, exhibited substantially decreased histone chaperone activity *in vitro* and histone mobility *in vivo* [14]. The human FANCD2 R302W mutation, in which the Arg302 residue is replaced by Trp, has been identified as an FA-associated mutation [52]. The cFANCD2 R305W mutant is proficient in both the ID complex formation with cFANCI and monoubiquitination in the cFANCI-dependent manner [14], suggesting that the defective histone chaperone activity of the mutant may be responsible for the FA pathogenesis. The cFANCD2 L234R mutant also exhibited defective histone chaperone activity *in vitro*. However, unlike the cFANCD2 R305W mutant, it is markedly defective in both the ID complex formation and monoubiquitination (Figs. 1, 2, and 3). The *in vivo* complementation assay using the FANCD2^−/−^ DT40 cells also revealed the difference between the cFANCD2 L234R and R305W mutants. The cFANCD2 L234R-expressing cells are quite sensitive to a DNA crosslinking agent, cisplatin (Fig. 1C). On the other hand, the cFANCD2 R305W mutant partially complements the DNA-damage sensitive phenotype of the FANCD2^−/−^ DT40 cells [14]. Therefore, the cFANCD2 L234R mutation affects the FANCD2 function more severely than the R305W mutation, both *in vitro* and *in vivo*.

## Supporting Information

S1 Figure **The full-length gel images for** **Fig. 1B and 1C** **.** (A, B) Cells expressing the GFP-fused wild type (WT) cFANCD2 or cFANCD2 L234R were treated with or without mitomycin C (MMC), and the WCEs were analyzed by western blotting using anti-FANCD2 (left panel) and anti-FANCI antibodies (right panel). S-forms and L-forms indicate non-ubiquitinated forms and monoubiquitinated forms of cFANCD2 and cFANCI, respectively. Non-specific bands are marked by the asterisks. (C, D) Cells expressing GFP-fused WT cFANCD2 or cFANCD2 L234R were treated with or without MMC, and the chromatin fractions were analyzed by western blotting using anti-FANCD2 (left panel) and anti-FANCI antibodies (right panel). “S” indicates non-ubiquitinated forms of cFANCD2 and cFANCI, and “L” indicates monoubiquitinated forms of cFANCD2 and cFANCI. Asterisks indicate the degradation products of GFP-cFANCD2 generated during the chromatin preparation.
(TIF)

S2 Figure **The full-length gel images for** **Fig. 3** **.** (A, B) Gel filtration analysis of the ID complex formation. Fraction numbers are shown in the bottom of gel-filtration profiles, and fractions indicated above the gel images were analyzed by 7% SDS-PAGE, and proteins were visualized by silver staining. Markers are indicated as ‘M’. Experiments with wild type (WT) cFANCD2 and WT cFANCI (A), cFANCD2 L234R and WT cFANCI (B). (C, D) Immunoprecipitation analysis of the cFANCD2 and cFANCI interaction. The cell extracts from chicken DT40 cells expressing GFP-WT cFANCD2 or cFANCD2 L234R were immunoprecipitated with an anti-GFP antibody. The immunoprecipitates were analyzed by western blotting using anti-chicken FANCD2 (C) and FANCI antibodies (D).
(TIF)
