# Supplementary figures and images for: Defective FANCI Binding by a Fanconi Anemia-Related FANCD2 Mutant
Source: PLoS One. 2014 Dec 9;9(12):e114752. doi: 10.1371/journal.pone.0114752 (PMC4260917; doi:10.1371/journal.pone.0114752)

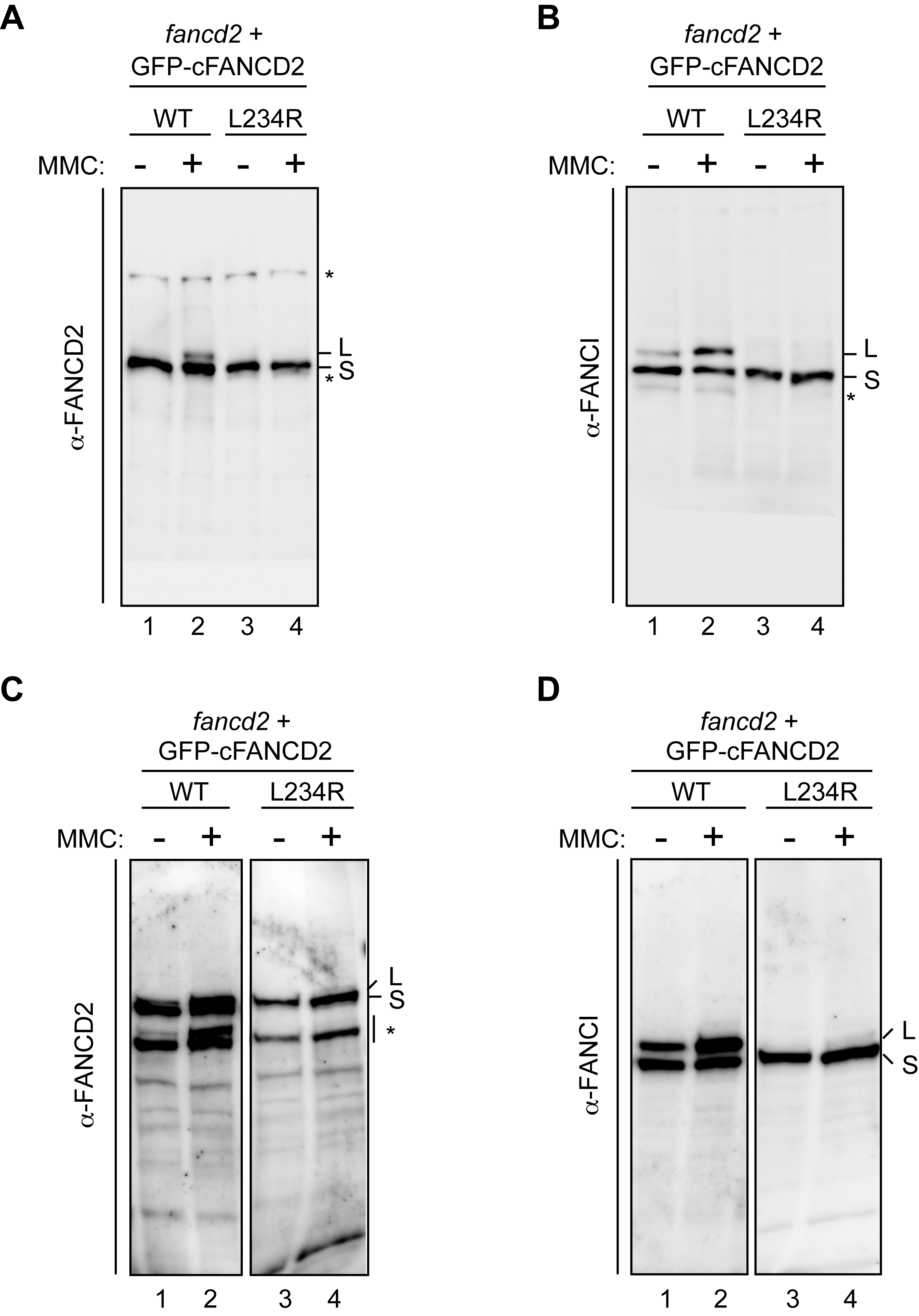

Supplement: S1 Figure — The full-length gel images for Fig. 1B and 1C . (A, B) Cells expressing the GFP-fused wild type (WT) cFANCD2 or cFANCD2 L234R were treated with or without mitomycin C (MMC), and the WCEs were analyzed by western blotting using anti-FANCD2 (left panel) and anti-FANCI antibodies (right panel). S-forms and L-forms indicate non-ubiquitinated forms and monoubiquitinated forms of cFANCD2 and cFANCI, respectively. Non-specific bands are marked by the asterisks. (C, D) Cells expressing GFP-fused WT cFANCD2 or cFANCD2 L234R were treated with or without MMC, and the chromatin fractions were analyzed by western blotting using anti-FANCD2 (left panel) and anti-FANCI antibodies (right panel). “S” indicates non-ubiquitinated forms of cFANCD2 and cFANCI, and “L” indicates monoubiquitinated forms of cFANCD2 and cFANCI. Asterisks indicate the degradation products of GFP-cFANCD2 generated during the chromatin preparation. (TIF) [file pone.0114752.s001.tif]

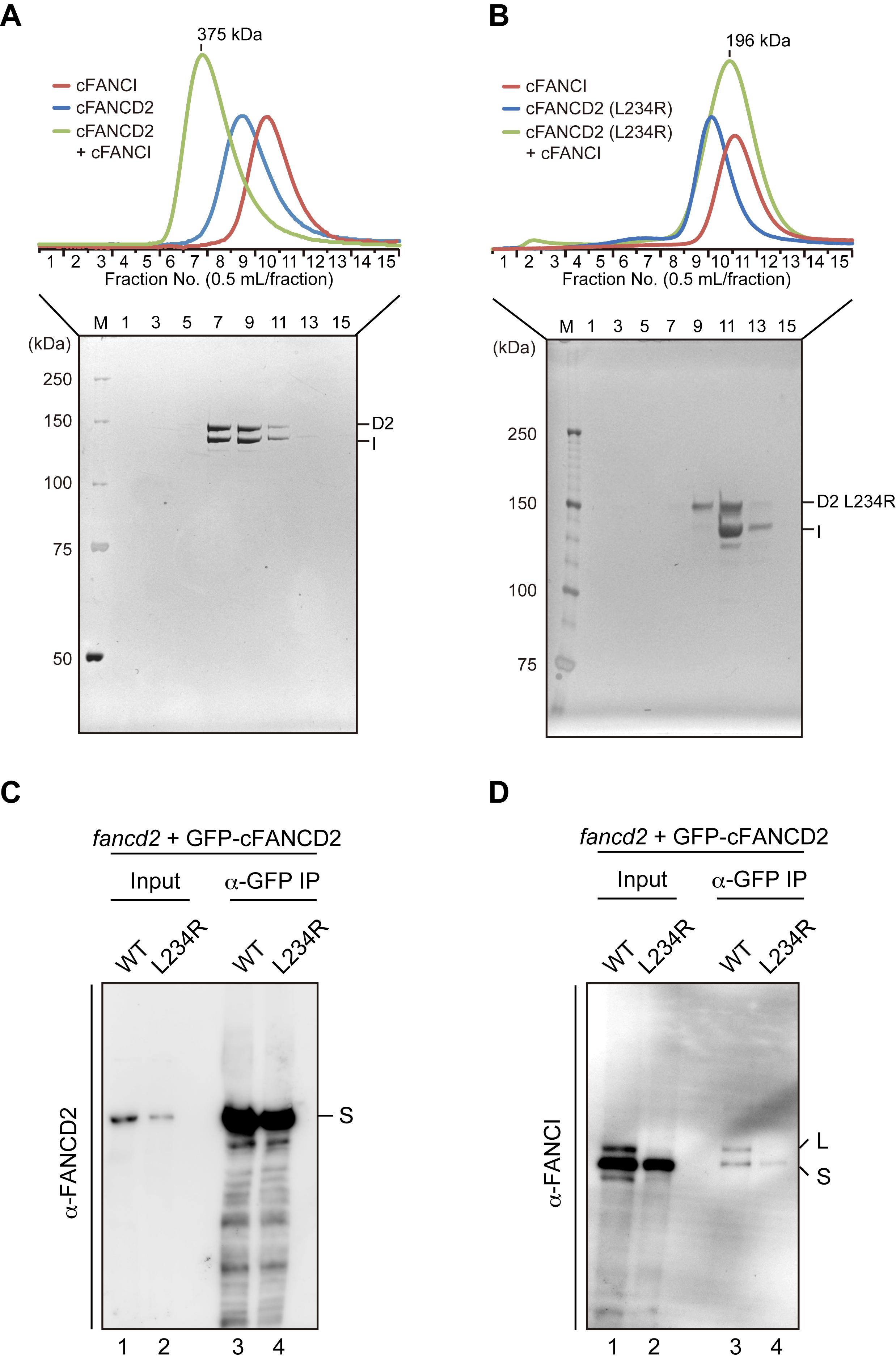

Supplement: S2 Figure — The full-length gel images for Fig. 3 . (A, B) Gel filtration analysis of the ID complex formation. Fraction numbers are shown in the bottom of gel-filtration profiles, and fractions indicated above the gel images were analyzed by 7% SDS-PAGE, and proteins were visualized by silver staining. Markers are indicated as ‘M’. Experiments with wild type (WT) cFANCD2 and WT cFANCI (A), cFANCD2 L234R and WT cFANCI (B). (C, D) Immunoprecipitation analysis of the cFANCD2 and cFANCI interaction. The cell extracts from chicken DT40 cells expressing GFP-WT cFANCD2 or cFANCD2 L234R were immunoprecipitated with an anti-GFP antibody. The immunoprecipitates were analyzed by western blotting using anti-chicken FANCD2 (C) and FANCI antibodies (D). (TIF) [file pone.0114752.s002.tif]
